# Characterization of 954 bovine full-CDS cDNA sequences

**DOI:** 10.1186/1471-2164-6-166

**Published:** 2005-11-23

**Authors:** Gregory P Harhay, Tad S Sonstegard, John W Keele, Michael P Heaton, Michael L Clawson, Warren M Snelling, Ralph T Wiedmann, Curt P Van Tassell, Timothy PL Smith

**Affiliations:** 1 USDA-ARS-U.S. Meat Animal Research Center, Clay Center, NE 68901, USA; 2 USDA-ARS-Beltsville Area Research Center, Beltsville, MD, USA

## Abstract

**Background:**

Genome assemblies rely on the existence of transcript sequence to stitch together contigs, verify assembly of whole genome shotgun reads, and annotate genes. Functional genomics studies also rely on transcript sequence to create expression microarrays or interpret digital tag data produced by methods such as Serial Analysis of Gene Expression (SAGE). Transcript sequence can be predicted based on reconstruction from overlapping expressed sequence tags (EST) that are obtained by single-pass sequencing of random cDNA clones, but these reconstructions are prone to errors caused by alternative splice forms, transcripts from gene families with related sequences, and expressed pseudogenes. These errors confound genome assembly and annotation. The most useful transcript sequences are derived by complete insert sequencing of clones containing the entire length, or at least the full protein coding sequence (CDS) portion, of the source mRNA. While the bovine genome sequencing initiative is nearing completion, there is currently a paucity of bovine full-CDS mRNA and protein sequence data to support bovine genome assembly and functional genomics studies. Consequently, the production of high-quality bovine full-CDS cDNA sequences will enhance the bovine genome assembly and functional studies of bovine genes and gene products. The goal of this investigation was to identify and characterize the full-CDS sequences of bovine transcripts from clones identified in non-full-length enriched cDNA libraries. In contrast to several recent full-length cDNA investigations, these full-CDS cDNAs were selected, sequenced, and annotated without the benefit of the target organism's genomic sequence, by using comparison of bovine EST sequence to existing human mRNA to identify likely full-CDS clones for full-length insert cDNA (FLIC) sequencing.

**Results:**

The predicted bovine protein lengths, 5' UTR lengths, and Kozak consensus sequences from 954 bovine FLIC sequences (bFLICs; average length 1713 nt, representing 762 distinct loci) are all consistent with previously sequenced mammalian full-length transcripts.

**Conclusion:**

In most cases, the bFLICs span the entire CDS of the genes, providing the basis for creating predicted bovine protein sequences to support proteomics and comparative evolutionary research as well as functional genomics and genome annotation. The results demonstrate the utility of the comparative approach in obtaining predicted protein sequences in other species.

## Background

Numerous whole genome sequence projects have been completed or are in progress, spanning a wide range of species among different orders. The genome sequences are providing novel insights into evolution and gene regulation that would have been impossible without these large-scale sequencing efforts. While a variety of sequencing strategies have been applied, the most common currently in use and the strategy chosen for the bovine genome relies mainly on whole genome shotgun (WGS) sequencing and assembly of the sequencing reads based on sequence similarity overlap. The bovine assembly will be supplemented by a much lower coverage of sequence from large-insert clones (Bacterial Artificial Chromosome, BAC) to provide connections between non-overlapping sequence contigs that represent chromosomal locations in close proximity to one another. A more comprehensive build of the genome sequence adds information from physical and genetic maps to WGS and BAC sequence to order contigs on a larger scale. An intermediate level of resolution and a critical check on the accuracy of the other methods can be provided by determining if the proper orientation, order, and spacing of exons in known expressed genes are maintained in the build. This approach requires knowledge of expressed transcript sequence to compare to the genome build.

Another use of transcript sequence is in annotation, a key to the utility of whole genome sequencing. Previous full-length cDNA sequencing projects have established the importance of experimentally derived mRNA sequences to produce gene models that establish accurate exon-intron boundaries [1-5]. These projects provided vital information about alternate splice forms of gene products that generate variation in form and function thought to be a key contributor to diversity in expression and phenotype. FLIC sequences also assisted in discriminating between alternative splicing and gene duplication or pseudogenes, a procedure that is difficult and error prone if based solely on clustered EST sequences.

The other main use of FLIC sequences has been generation of predicted protein sequence, providing a resource to support proteomic approaches and comparative analysis to reveal details of protein function. This goal requires accurate reconstruction of CDS portions of the bona fide transcripts expressed in the target tissues, which may be problematic with clustered EST as mentioned above.

The present effort was undertaken to support all of the potential uses of bFLIC data. The International Bovine Genome Sequencing Consortium [6] led by Baylor College of Medicine recently released the second, 6-fold coverage genome assembly (Worley, K. personal communication). Refinement of the assembly will be facilitated by incorporating bFLICs in the gene modeling and assembly process, similar to their utility in the assembly of genomes of other organisms. The bFLICs will also support efforts at NCBI and ENSEMBL to derive accurate gene models, and derive predicted protein sequence databases. In this sense, the present study is similar to previous full-length cDNA projects carried out for humans [1], mice [3], and other species [5,7]. However, a different approach was used to generate the data than in previously described efforts, as the first step of this project employed sequencing of pooled-tissue, normalized libraries [8,9] that had not been constructed by procedures to enrich for full-length clones, since such procedures could potentially introduce bias that would decrease the diversity of observed mRNA. Moreover, a primary goal of the project was to develop a method to consistently select full-CDS clones from these libraries based on comparison of the single-pass, 5' end sequences to the human Reference Sequence [10] (RefSeq) mRNA database.

This report characterizes the sequences of bovine full-CDS clones selected with a method using 5' end EST sequence data as input. This method efficiently identified apparent bovine homologs of human RefSeq mRNA sequences, collected the full insert sequence, and annotated the resulting bFLICs with GeneIDs, product, repetitive elements, and predicted protein sequences. The method described should be particularly useful for generating full-CDS and predicted protein sequences for organisms with mature databases of sequence from other species in the order (e.g. other mammals) but not included in complete genome sequence projects. The success of the method was characterized by comparison of the bFLIC sequences to human Refseq mRNA and mammalian UTRdb, [11]. Because the investigation was initiated prior to release of the assembled bovine genome, direct comparison between bovine genomic and bFLIC sequence was problematic.

Without available genomic or full-CDS cDNA sequence, it is common practice to rely on gene clusters such as Unigene [12] or TIGR Gene Indices [8,9,13,14] for transcript predictions. These computational derived consensus assemblies containing open reading frames (ORFs) are generated from single pass reads through cDNA libraries. These clusters provide a very important resource for putative gene models and products. The TIGR *Bos taurus *Gene Index *(Bt*GI) was compared to bovine full-CDS sequences to confirm the existence of experimentally determined transcripts in the computed clusters. This characterization of gene clusters to full-CDS sequences may assist investigators to interpret the significance of their searches against gene cluster databases.

## Results and Discussion

### Strategy for bovine full-CDS selection and sequencing

**Figure 1:**
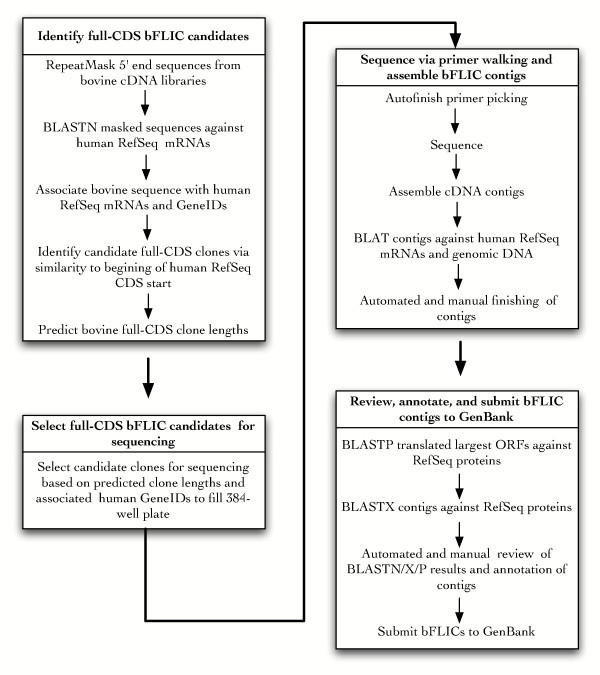
**Scheme for bovine full-length insert cDNA (bFLIC) sequence production**. Overall scheme for selecting, sequencing, and annotating bFLIC clones. If the largest ORF of the bFLIC spans the CDS of a human RefSeq transcript, then this clone is designated a full-CDS bFLIC with "complete cds" in the definition line in the GenBank submission.

The overall strategy for bFLIC processing is depicted in Figure 1 and is similar to an approach recently described for chicken bursal lymphocytes[15]. Single pass 5' reads from bovine clones from five pooled-tissue, normalized EST libraries [8,9] were compared to human RefSeq transcripts to identify potential full-CDS clones so each selected clone was associated with a human RefSeq and GeneID. These EST libraries were chosen because they were generated by the USDA labs collaborating on this project, so the clones were readily obtainable, and they represented over 70% of the total EST sequences in GenBank at the time the project was initiated.

The majority of clones were selected to represent unique loci as defined by human GeneID, and in cases where multiple EST clones were available for a given GeneID the clone with the longest predicted clone length was chosen. Additional criteria were also used relative to the predicted length of insert based on human cDNA length, in order to avoid clones of relatively short insert length. Specifically, clones were selected in size categories between 1,000 and 5,000 bp. A minority of clones were then chosen that were redundant to previously targeted GeneID to ascertain the impact of alternative splicing on EST cluster-based sequence databases. This clone selection yielded full-CDS bFLICs cDNAs with 80% efficiency, which was limited in part by the method of library construction that incorporated a digestion with restriction enzyme *NotI *following second-strand cDNA synthesis to generate a compatible cloning site on the 3' end of the cDNA [8,9]. Of the 20% failures, 45% are due to *NotI *sites within the transcript sequence that caused premature termination of the cDNA representations of the transcripts. This is a much higher rate than anticipated based on the average occurrence of *NotI *sites in genomic DNA and probably reflects a higher percentage of cytidine (C) and guanosine (G) in mRNA sequence (the recognition site for *NotI *is GCGGCCGC). Hopefully, recent advances in cDNA library production that avoid this type of difficulty will reduce the incidence of truncated clones in future efforts.

Putative full-CDS FLICs selected were sequenced with a "primer walking" procedure in which each sequence read was used to design a primer to extend sequence in the 3' direction. The reads were assembled into contigs, screened for polyA tail and vector, and compared to the human RefSeq transcripts after every walk. Once the 3' end of the insert was encountered (polyA tail or vector), the contig was manually checked for low quality base calls; 5' and 3' finishing primers were used to improve these low quality regions before they subjected to annotation. For each bFLIC, the translated longest ORF (putative protein coding sequence) of the bFLIC was compared to the RefSeq protein database using BLASTP. The bovine protein-human protein comparison served as consistency check with respect to the annotators' association of the bFLIC to human RefSeq. The bFLIC nucleotide sequence comparison to human RefSeq protein sequence (BLASTX) exposed potentially artificial frameshifts/insertion/deletions if present. Only when there was agreement between the annotators' annotation and the computational comparisons were the bFLICs submitted to GenBank.

### Summary and length distributions of the bFLICs

**Figure 2:**
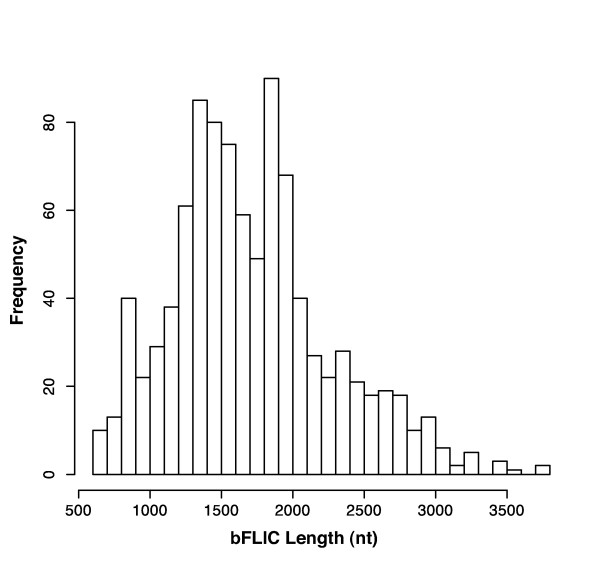
**Distribution of bFLIC lengths**. Clone lengths targeted were 1000 +/- 200, 1500 +/- 200, 2500 +/- 200, and 3000 – 4000 nucleotides (nt).

Figure 2 shows the distribution of bFLIC clones with mean 1713 nt (s.d. = 557) with values ranging from 605 to 3767 nt. This multi-modal distribution reflects the non-random selection criteria employed. Predicted clone lengths targeted were 1000 +/- 200, 1500 +/- 200, 2500 +/- 200, and 3000 – 4000 nt. This histogram shows that bFLICs larger and smaller than 2000 nt can be successfully sequenced. The bFLIC data is summarized in Table 1. The bFLICs have been used as the source sequence for 411 bovine RefSeqs for annotating and assembling the NCBI build of the bovine genome.

**Table 1:** Summary of full-CDS bFLICs

Number bFLICs submitted	954
Number unique loci	762
Average length (nt)	1713
Success rate (number full-CDS Sequence/number clones sequenced)	80%
Number *Bt *full-CDS bFLICs used as source clones for GenBank *Bt *gene models (Entrez Gene)	411

### Comparison of bFLICs to human RefSeq mRNA and protein

**Figure 3:**
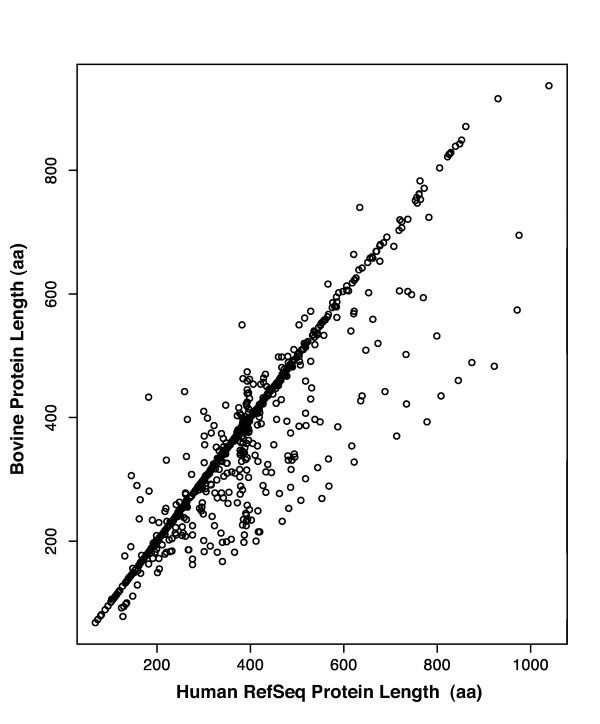
**Predicted bovine protein length vs. human protein length**. Comparison of the predicted proteins from 954 full-CDS bFLICs vs. their human RefSeq protein homologs, unit is amino acid (aa).

**Figure 4:**
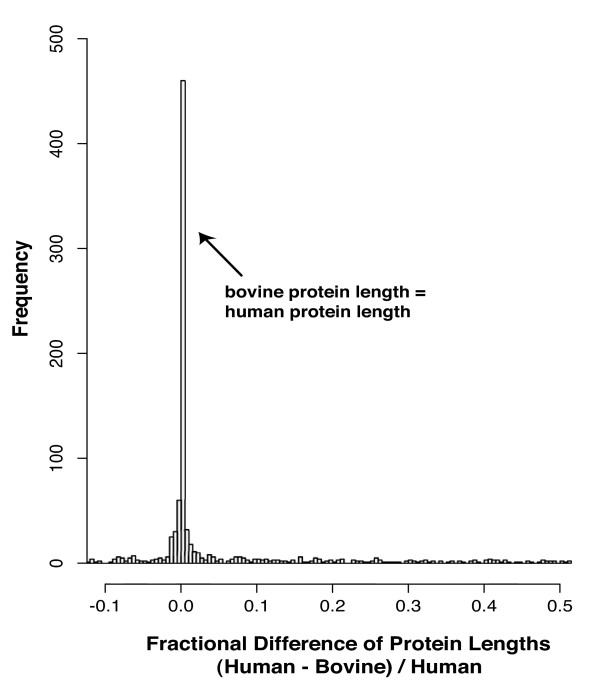
**Distribution of predicted bovine protein lengths vs. fractional difference from human protein homologs**. The frequency of occurrence of bovine protein lengths vs. the fractional difference of protein length from human homolog, (human - bovine)/human.

The protein sequence lengths translated from the full-CDS bFLIC CDSs range in length from 68 to 937 amino acids (aa) (Figure 3). In general, the bovine proteins lengths are similar to that of their human homologs. The relationship between homologous bovine and human proteins is demonstrated in Figure 4, where the distribution of bovine protein lengths is plotted versus their fractional difference from human protein homolog lengths. Figure 4 shows that the most common occurrence is when the bovine and human protein homolog lengths are the same, this occurs with 44% of bovine full-CDS clones. Seventy-five percent of the bovine full-CDS clones code for proteins within +/- 7% their human homolog protein lengths. Bovine proteins that are shorter than their human homologs constitute 34 % of our submission, while those that are longer constitute 22 %. These results show that while 75% of the bFLICs code for proteins identical or nearly identical in length to their human homologs, the remaining bFLICs tend to be shorter than their human homologs rather than longer. Comparison of the "short" bFLICs to the human genome and message sequence show reveals no obvious preference for internal vs. 3' terminus exon excision/change in the "short" bFLICs. The tendency towards shorter bFLICs may be due to a cloning bias towards shorter inserts resulting in the selection and sequencing of shorter bFLIC isoforms. Alternatively, this tendency may reflect fundamental differences in gene structures between human and cattle orthologs and/or paralogs. The possibility that some of these short bFLICs are associated with pseudogenes cannot be eliminated.

### Comparison of bFLICs 5' UTR to mammalian 5' UTR – verifying CDS start statistics

**Figure 5:**
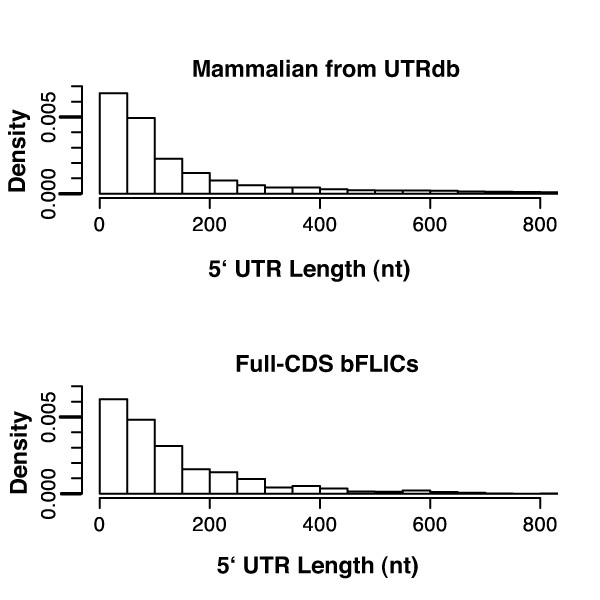
**Comparison of the 5' UTR lengths distribution between mammalian transcripts and bFLICs**. Distribution in 5'UTR lengths (nucleotide) from the 6262 5' UTRs in UTRdb (release 16) and 954 bFLIC 5' UTRs. Density is calculated so that the integrated area of all of the bins for each distribution is equal to 1.

The differences in bovine protein length relative to their human homologs could be an indication of systematic errors in the clone picking algorithm, sequencing, or annotation procedures. Since the bovine clones were selected to have a high degree of homology within the region of the human message surrounding its initiation codon ATG, differences in clone length should be attributable to truncation/extension of the CDS and differences in the 3' and 5' untranslated region (UTR). The incorrect determination of CDS start in the clone selection step, sequencing errors generating frameshifts and/or insertions/deletions, and misidentifying CDS start in the annotation process could all contribute to the misidentification of the extent of CDS, and by inference, the 5' UTR. Comparisons between full-CDS bFLIC and mammalian 5' UTR length distributions would show a bias towards larger or smaller bovine 5' UTR if the bovine CDS start was systematically chosen too far upstream or downstream of its actual position. Figure 5 shows that bovine and mammal 5' UTR length distributions are very similar throughout the range of 5'UTR lengths. Because only 954 sequences were sequenced, relatively few bovine full-CDS clones were found with 5' UTR lengths > 300 nt. This comparison indicates that start methionines weren't systematically misidentified skewing the 5' UTR lengths, but rather, is consistent with previously annotated 5' UTR mammalian sequence.

### Comparison of bFLICs to mammalian Kozak consensus sequences

**Figure 6:**
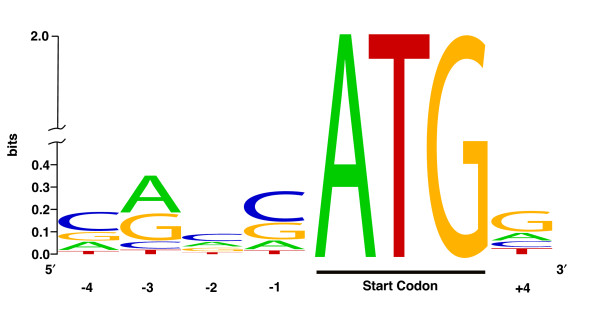
**Kozak consensus sequence surrounding bFLIC start methionine**. Kozak consensus sequence surrounding bovine start methonine using WebLogo [29].

The vertebrate initiation codon context is (A/G)CCATGG [16,17], with the initiation ATG codon underlined. The consensus sequence in Figure 6 shows that the most highly conserved position is 3 nucleotides upstream from the start codon. This consensus sequence exhibits the expected behavior, with the most highly conserved position, being an A, 3 nucleotides upstream from the start codon at position -3. The comparison of bovine consensus start logo to the human consensus start logo in Iacono et al. [18] reveals a high degree of similarity. This comparison shows that although there is less conservation at positions -3 and -2 in cattle, there is roughly equal conservation at positions -1 and +4 in cattle and human. Moreover, the relative preference for every nucleotide base from positions -3 to +4 is identical between cattle and human. This high degree of similarity may be surprising, especially since the Kozak sequence is not strictly conserved in eukaryotic mRNAs [19]. Bovine clones were selected for sequencing based on their close homology to human near the CDS start, so it shouldn't be the surprising that sequences were obtained that were similar to human near the CDS start. The conservation of the bovine Kozak consensus sequence suggests that, as with the 5' UTR analysis, start methionines weren't systematically misidentified, but rather, is consistent with previously annotated human transcripts.

### Alternative splicing

**Figure 7:**
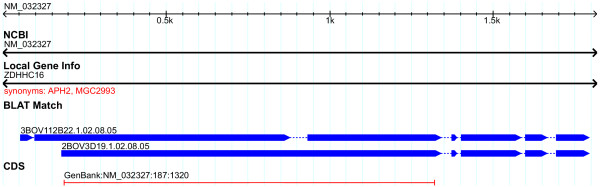
**Putative alternative splicing in bFLICs – bovine transcripts vs. human transcript**. Alignment of 3BOV112D22 (BT021708) and 2BOV3D19 (BT021853) full-CDS bFLICs to the human RefSeq transcript NM_032327 of the ZDHHC16 gene.

**Figure 8:**
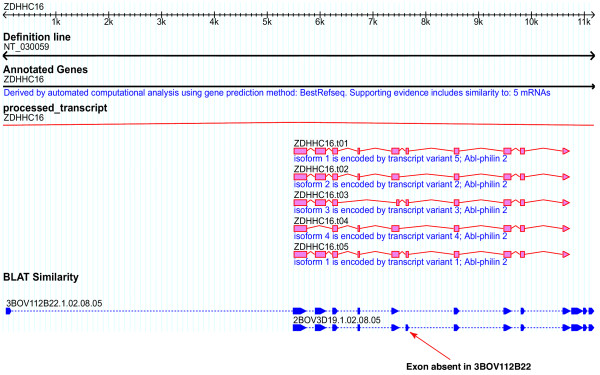
**Putative alternative splicing in bFLICs – bovine transcripts vs. human genome**. Alignment of 3BOV112D22 (BT021708) and 2BOV3D19 (BT021853) full-CDS bFLICs against human genomic (and splice forms) where an extra exon in the CDS is present in 2BOV3D19 that is absent from 3BOV112D22.

Multiple clones were selected for 92 loci, ranging from 12 clones for a single locus (COMMD4 GeneID:54939) to 2 clones for 51 loci. Comparison of full-CDS bFLICs to human message, protein, and genomic suggests alternative bovine transcripts exist for five loci, PSMD4 (GeneID:5710), BCL2L14 (GeneID:79370), NME7 (GeneID:29922), ZDHHC16 (GeneID:84287), HYAL1 (GeneID:3373). Figure 7 shows the comparison of the 3BOV112D22 (BT021708) and 2BOV3D19 (BT021853) to human RefSeq NM_032327. This shows a gap in the coverage of 3BOV112D22 on the human RefSeq CDS while 2BOV3D19 completely covers the human CDS. In Figure 8, where the full-CDS bFLICs are compared to human genomic, it is observed that an exon is present in 2BOV3D19 that is absent in 3BOV112D22. Alternate splicing has been observed for these five loci in humans.

### Comparison of bFLICs to TIGR BtGI

The sequences from all EST libraries used for this study have been previously incorporated into the TIGR *Bt*GI. This presented an opportunity to verify the TCs (Tentative Consensus sequences) constructed with single pass reads of source clones by comparing them to contigs built from multi-pass full-length sequencing of the same source clones.

The TCs of TIGR *Bt*GI (Release 11, September 28, 2004) were compared to the full-CDS bFLICs using BLAT[20]. A threshold of 300 or more identities, 1/2 the size of our shortest bFLIC, was chosen to minimize short matches. After the identities threshold was applied, a total of 1346 distinct TCs were found to be similar to 933 of the original 954 bFLICs. If only bFLICs that are members of TCs were considered, 1250 TCs were found to be similar to 855 distinct bFLICs. If there was a further constraint that only matches between a (query) TC and it's (subject) member source clones be considered, then 740 distinct TCs were found to be similar to their source member bFLICs. In the latter analysis, 1 TC can match multiple bFLICs, but not vice versa. This number is quite close to the 762, the number of distinct loci associated with our 954 bFLICs generated in the annotation pipeline. 92 full-CDS bFLICs are not members of a TC.

The analysis of the BLAT similarities between the TIGR *Bt*GI and bFLICS is complicated by the fact that because multiple TCs can represent a single locus by virtue of alternative splice forms, mis-assembly, or other aspects of shared gene structure, a single bFLIC may be similar to multiple TCs besides its parent TC. Accordingly, the BLAT analysis was segregated into two groups. The first group (A) was the comparison of the bFLICs to all 40,810 TCs, where in general, and given our BLAT threshold, a bFLIC will be similar to more than 1 TC. This comparison results in BLAT hits to 1346 TCs. The second group (B) was a comparison of 855 full-CDS bFLICs to only those TCs that the bFLICs are members of, a smaller set (740) of TCs than the first group. Group B TCs represent the minimum number of TCs that span the "transcription potential" of 855 bFLICs.

Table 2 show that complete or nearly complete fractional coverage (> = .95) of a bFLIC by a single TC was observed for nearly 1/2 of the bFLICs relative to groups A and B. Relaxing the fractional coverage requirement to determine the number of bFLICs that have .95 or less TC fractional coverage, exposes a significant difference between group A and B. In group A, 155 bFLICs (out of 933) are shared between the > = .95 and < .95 fractional coverage levels accounting for the distribution of bFLICs between the two levels (672 + 414 - 153 = 933). This is not unexpected as a TC that exhibits > = .95 fractional coverage from one bFLIC, may also cover at < .95 for another bFLIC. In group B, only 2 bFLICs (out of 855) are shared between the > = .95 and < .95 fractional coverage levels, accounting for the distribution of each bFLIC (401 + 456 - 2 = 855) between these two levels. Group B provide a less redundant description of the transcript space of the bFLICs. Unfortunately, when analyzing the sequence of non-TC member transcripts the analysis would typically be conducted in a group A manner. As the bFLIC fractional coverage level is reduced down from < .95 through < .15 level, the number of bFLIC meeting this requirement decreases. The process of decreasing the fractional coverage level amounts to finding bFLICs that aren't well represented by the TCs in the TIGR *Bt*GI. As the fractional coverage level decreases, the bFLICs expectedly tend to lengthen. The data in Table 2 shows that about 1/2 the bFLICs are represented well by single TCs in the TIGR *Bt*GI using strict similarity criteria, but using less strict similarity criteria will result in a more ambiguous representation of the bFLIC using TCs because of the similarity between TCs. As average bFLIC lengths increase from about 1500 nucleotides, the probability decreases that a single TC matches with high fidelity an entire bFLIC.

**Table 2:** BLAT Results: TIGR *Bt*GI TCs vs. full-CDS bFLICs with identities > = 300

	**A**		**B**	
	
	No bFLIC TC membership requirement		bFLIC required to be member in query TC	
	
	933 total bFLICs in all alignments		855 total bFLICs in all alignments	
Fractional coverage of bFLICs by any single TC	Number of bFLICS	Average Contig Length	Number of bFLICS	Average Contig Length

> = .95	414	1470	401	1474

< .95	672	1782	456	1865
< .90	639	1797	409	1909
< .80	615	1810	360	1964
< .50	531	1846	264	2081
< .25	389	2139	163	2484
< .20	97	2430	29	2664
< .15	29	2781	8	2920

In order to quantify how well the TCs cover the bFLICs, the number of bFLICs matched by TCs at different fractional coverage levels was determined. Fractional coverage of a bFLIC by a TC = number of identities in the BLAT match/bFLIC length.

Single pass 5' and 3' reads for 169 full-CDS bFLICs were previously incorporated into the TIGR *BtGI*. The 5' and 3' single pass reads for 94 (56%) were assembled into the same TCs, while 75 (44%) single pass end reads were placed in different TCs. Using the admittedly limited dataset of 169 bFLICS, it is observed that about 1/2 of the TCs were self-consistently constituted from their source clone sequences. It is likely that the TCs not self-consistently constituted were assembled without adequate data linking the two ends from the single source clone.

## Conclusion

The bovine transcript sequences described here presently represent the largest publicly accessible resource of annotated full-CDS bFLICs. [*Note added during review*: since this manuscript's submission, 1710 bovine full-length insert cDNA sequences have been submitted to the Mammalian Gene Collection at NCBI by the Bovine Genome Sequencing Program, Genome Sequence Centre, BC Cancer Agency, Vancouver, BC, Canada] The comparative genomics approach employed for clone selection and the database driven sequencing and analysis pipeline provides a mechanism to target and produce full-CDS bFLICs for specific loci that are represented in available cDNA libraries. The full-CDS bFLICs are being incorporated in the NCBI build of the bovine genome. The approach described here should be adaptable for producing full-CDS FLICs for other organisms, and is particularly appropriate for those organisms without available FLIC sequences but with 5' end EST sequences. Analysis of the bFLICs with respect to TIGR *Bt*GI shows that about 1/2 the bFLIC sequences are well represented by the TCs, while a smaller fraction of the remaining bFLIC aren't well represented by any single TC. Smaller bFLICs are more easily represented by a single TC in the TIGR *Bt*GI than longer bFLICs. As TCs grow larger than 1500 nucleotides, they become increasingly dissimilar from their FLIC counterparts, and therefore become increasingly less suitable as evidence for basing an accurate gene model on. The full-CDS bFLIC Kozak consensus sequence and 5' UTR length distribution is consistent with prior human and mammal transcript data. The genome complexity exhibited by the alternatively spliced bFLICs correspond to human loci that also exhibit alternatively spliced human transcripts. These results only hint at the bovine genome's inherent complexity. These bFLICs and their annotations provide a significant starting point to investigate the bovine genome and gene expression.

## Methods

### Clone selection

A total of 195,443 5' end sequence reads from the 1BOV, 2BOV, 3BOV, 4BOV, and 5 BOV [8,9] cDNA libraries were masked for repeats with RepeatMasker[21] and compared to human RefSeq mRNA using BLAST [22] yielding 146,741 distinct bovine clones with BLAST hits, 116,911 of which had 300 or more bases with phred quality score greater than or equal to 20. The bovine cDNAs were associated with the human RefSeq mRNAs with the highest bit score, and through the RefSeqs, the bovine cDNAs were associated with human GeneIDs. Based on the clone sequence similarity to the beginning of CDS of human RefSeq mRNAs, 9,989 potential full-CDS clones were identified, associated with 3,482 distinct human loci. Predicted clone length was defined by the sum of the length of the bovine clone upstream of the beginning of the BLAST match plus the length of the human RefSeq sequence downstream from the beginning of the BLAST match. The putative full-CDS clones were grouped by loci (human GeneID) and predicted clone length.

Clones were loaded in 384 well plates and sequenced via primer walking, using a combination of automatically generated primers from autofinish [23], or manually generated primers from consed [24]

### Primer walking

The clones were sequenced, assembled, and annotated in a semi-automated pipeline involving a database that stored and provided sequencing, primer, and annotation information for every clone. Perl scripts were used to process reads, place them in the appropriate directories, instantiate phredPhrap for contig assembly, automate the detection of polyA, vector, pick walking primers and update the database. bFLIC clones were sequenced 5' -> 3' until polyA or vector was encountered. In regions of low quality sequence, reverse read primers were manually picked. Perl scripts were also use to automate BLAT[20] comparisons of bFLICs with human RefSeq mRNAs.

### Annotation

Human annotators assigned bFLICs to human RefSeq mRNA homologs and determined whether or not the bFLIC was a full-CDS clone. Gbrowse [25] was used to display bovine/human alignments and Artemis [26,27] was used for manual annotation of sequence when required. A clone was deemed to be full-CDS if the BLAT query bFLIC region encompassed the entire CDS of its human homolog, and/or the BLAT query region encompassed the beginning of the human homolog's start methionine and exhibited a polyA stretch of at least 13 adenosines on the 3' end. Subsequently, each masked bFLIC was processed through the quality check/assurance portion of the pipeline where the largest translated ORF was compared with BLASTP [22] to human RefSeq proteins and the entire nucleotide sequence of the bFLIC was compared to RefSeq proteins with BLASTX [28]. A bFLIC was flagged for GenBank submission only if the highest scoring BLASTX and BLASTP hits originated from the same RefSeq mRNA and was identical to the transcript assigned through human review.

#### RepeatMasker parameters

-species cow -xsmall. Repeats are masked with lower case letters using the cattle specific repeat library.

#### BLASTX parameters

-U -F "m S" -I T -f 14 -e 1e-20 -a 2 -b 15. Use RepeatMasker output as input. Allow for extension through repetitive regions, but alignment isn't seeded in repetitive region (soft masking).

#### BLASTP parameters

-v 1 -b 1 -f 14 -e 1e-20 -a 2

The GenBank Accessions for the bFLIC clones are: [GenBank:BT020623 GenBank:BT021084, GenBank:BT021145 ... GenBank:BT021203, GenBank:BT021479 ... GenBank:BT021911].

### Kozak consensus sequence

The sequence spanning from 6 nucleotides upstream to 3 nucleotides downstream of the adenosine of the start ATG was extracted from each bFLIC and aligned with clustalw. The alignment file was used as input to WebLogo.

## Authors' contributions

GPH designed the full-CDS database, developed and implemented algorithms for full-CDS FLIC detection and scripts for the sequencing, analysis, annotation and submission pipelines. TSS performed FLIC sequencing and annotation. MPH and MLC assisted in the annotation of the bFLICs as well as suggesting improvements in the pipeline. WS assisted in running sequence comparisons on a compute cluster, and perl script development. RW assisted in masking repeats. KV assisted in FLIC sequencing. JWK assisted in the design of the full-CDS database and FLIC pipelines. TPLS contributed to all phases of algorithm, database and pipeline development as well as overseeing FLIC sequencing.
